# Medical Attendance on Families by the Year

**Published:** 1852-02

**Authors:** 


### MEDICAL ATTENDANCE ON FAMILIES BY THE YEAR.

We heartily approve of every word in the following paragraph, which we copy from the *New Orleans Medical and Surgical Journal.* We had supposed that the reprehensible practice to which it refers had fallen into such disrepute, that but very few respectable physicians were guilty of it, and are sorry to learn from the New Orleans editor that it is on the increase in any part of the country. A practice more injurious to the profession we cannot easily conceive of. If among our readers there is a practitioner in the habit of thus hiring his services out by the year, we beg him to think seriously of what would be the consequence should his example be generally imitated? What would the practice of medicine be worth, if such a system of underbidding were to become common? If physicians could be induced to come into market as public contracts are let, how long before our noble profession would sink down to the level of the humblest trade? We confess we cannot understand how any man possessing proper self-respect could consent thus to cheapen his professional services. Better starve, we would say to our young brethren, than attempt to get into practice by underbidding. We have no idea that any physician ever rose, or ever can rise, to real eminence in his profession who has no juster estimate of its value and dignity than such a practice would imply. We commend the sentiments of our respected contemporary to any practitioners who may have been betrayed into it.

"Although condemned in strong terms and unequivocal language by the National Medical Association, yet we regret to state, that the practice of attending families and individuals by the year, has become crying—a great evil, in many of our cities, if we are correcily informed. It is unjust in itself, and cannot result in any thing but mischief to the profession and to the parties contracting. The physician should receive a fair—a just remuneration for his services; and his clients should be compelled to pay only for such professional attention as they may receive; but "yearly practice" aims at a species of miserable, petty monopoly, which is at war with the objects—the noble purposes of a liberal and enlightened profession. You may bargain with your grocer, your butcher, your laundress, and no harm comes of or by it; but for an honorable, an educated physician, to hire himself by the year, like a slave; to pledge his talents and his services, for a stipulated sum, is in direct violation of the ethics of the profession, and indicates at once an unwillingness to enter the field of fair and honorable competition with our brethren."

